# Community Profile and Drivers of Predatory Myxobacteria under Different Compost Manures

**DOI:** 10.3390/microorganisms9112193

**Published:** 2021-10-21

**Authors:** Wei Dai, Ning Wang, Wenhui Wang, Xianfeng Ye, Zhongli Cui, Jieling Wang, Dandan Yao, Yuanhua Dong, Hui Wang

**Affiliations:** 1 CAS Key Laboratory of Soil Environment and Pollution Remediation, Institute of Soil Science, Chinese Academy of Sciences, Nanjing 210008, China; daiwei@issas.ac.cn (W.D.); nwang@issas.ac.cn (N.W.); wangjieling0407@126.com (J.W.); yaodandan@issas.ac.cn (D.Y.); yhdong@issas.ac.cn (Y.D.); 2 University of Chinese Academy of Sciences, Beijing 100000, China; 3 School of Life Sciences, Anhui Agricultural University, Hefei 230036, China; wangwenhui@ahau.edu.cn; 4 Key Laboratory of Agricultural Environmental Microbiology of Ministry of Agriculture, Nanjing Agricultural University, Nanjing 210095, China; T2019119@njau.edu.cn (X.Y.); czl@njau.edu.cn (Z.C.)

**Keywords:** myxobacteria, manure compost, community profile, predation bacterial

## Abstract

Myxobacteria are unique predatory microorganisms with a distinctive social lifestyle. These taxa play key roles in the microbial food webs in different ecosystems and regulate the community structures of soil microbial communities. Compared with conditions under conventional management, myxobacteria abundance increases in the organic soil, which could be related to the presence of abundant myxobacteria in the applied compost manure during organic conditions. In the present study,16S rRNA genes sequencing technology was used to investigate the community profile and drivers of predatory myxobacteria in four common compost manures. According to the results, there was a significant difference in predatory myxobacteria community structure among different compost manure treatments (*p* < 0.05). The alpha-diversity indices of myxobacteria community under swine manure compost were the lowest (Observed OTU richness = 13.25, Chao1 = 14.83, Shannon = 0.61), and those under wormcast were the highest (Observed OTU richness = 30.25, Chao1 = 31.65, Shannon = 2.62). Bacterial community diversity and Mg^2+^ and Ca^2+^ concentrations were the major factors influencing the myxobacteria community under different compost manure treatments. In addition, organic carbon, pH, and total nitrogen influenced the community profile of myxobacteria in compost manure. The interaction between myxobacteria and specific bacterial taxa (Micrococcales) in compost manure may explain the influence of bacteria on myxobacteria community structure. Further investigations on the in-situ community profile of predatory myxobacteria and the key microorganism influencing their community would advance our understanding of the community profile and functions of predatory microorganisms in the microbial world.

## 1. Introduction

Today, predation is considered a major evolutionary and ecological driver that can influence community structure and ecosystem function [1,2,3]. Although extensive research has been carried out on the predation behavior of large organisms such as animals and plants, predation is much less understood in the microbial world [3]. Myxobacteria are the first taxa of bacteria described as micro-predators, which are capable of secreting antibiotics, hydrolases, and bacteriolytic compounds to kill and lyse their prey microorganisms, including bacteria, fungi, protozoa, and other microorganisms [2,4,5,6]. Myxobacteria are reportedly highly adaptable cosmopolitans distributed in diverse environments, such as nutrient-rich soil, compost, sand, rocky soil, freshwater lake mud, and the sea [7,8,9,10]. However, our understanding of the factors driving the profile of predatory myxobacteria communities under different environmental conditions remains poor.

Interactions between myxobacteria and prey populations are a key aspect of the microbial food web [11], and predatory myxobacterium controls cucumber Fusarium wilt by regulating soil microbial community structure via decrease modularity and community number and increase connection number per node [12]. In addition, the myxobacteria *Corallococcus* sp. strain EGB can prey on diverse soil bacteria and could influence microbial community structure in a microcosm system [13]. The accumulation of prey-specific predacious genes in myxobacteria genomes partly explains the broad range of myxobacteria prey [14], which makes it reasonable to speculate that the bacterial community structure (composition and abundance) in microbial ecological niches could influence the community profile of myxobacteria.

In the Anthropocene, manure, which is mainly excreted by animals or derived from plant residues, is an environmentally friendly soil amendment used to manage soil degradation, and, in turn, increase crop production stability and agroecosystem functioning [15]. Manure application improves soil biochemical properties, such as soil organic carbon, total nitrogen (TN), and microbial biomass carbon [16]. Previous studies have demonstrated that myxobacteria naturally exist in compost manure [10,17,18] and that the diversity of soil microorganisms and myxobacteria increases under organic farming conditions [19,20]. In addition, environmental factors could influence the ecological distribution of myxobacteria in soil [21]; however, it is unclear whether the application of compost manure rich in myxobacteria would increase myxobacteria abundance in the soil. In addition, whether environmental factors directly influence the distribution of myxobacteria in compost manure, or indirectly influence myxobacteria distribution via their effects on prey diversity in compost require further investigations.

The present study aimed to investigate the factors driving myxobacteria community profile under four common compost manures. The specific objectives were (1) to investigate the community profile of predatory myxobacteria under different compost manures, (2) to explore the abiotic factors influencing the community profile of predatory myxobacteria in compost manure, (3) to explore the correlation between predatory myxobacteria diversity and bacterial community diversity under different compost manures, and (4) to explore the role of predatory myxobacteria in the bacterial community networks in compost manure and the correlations with associated bacterial groups.

## 2. Materials and Methods

### 2.1. Sample Collection and Analysis

Four types of compost manure were used in the analyses in the present study. Cow dung (CD, Qingshen Zhongxing Farming Professional Cooperative in Meishan city, Sichuan Province, China), swine manure (SM, Tenghui Farming in Yingtan city, Jiangxi Province, China), and chicken manure (CM, Longxiang Farming in Suqian city, Jiangsu Province, China) samples were collected from commercial composts produced by aerobic fermentation. Wormcast manure (WC) obtained by vermicomposting wormcast for one month, was collected from our laboratory (produced by earthworms ingesting cow dung samples). Each compost manure treatment included four replicates, and the different compost samples were divided into two parts, placed on ice, and transferred to the laboratory. One part was stored at −20 °C for use in DNA extraction and microbial analyses. The other part was stored at 4 °C for the determination of physicochemical properties. The pH, TN, organic carbon (OC), ammonium-nitrogen (NH_4_^+^-N), nitrate-nitrogen (NO_3_^−^-N), total potassium (TK), and total phosphorus (TP) were determined based on methods described in a previous study [19]. The exchangeable cations—Ca^2+^, Mg^2+^ and Na^+^ (1 M NH_4_-acetate pH 7.0, ICP-MS, PerkinElmer Optima, Waltham, Massachusetts, USA).

### 2.2. DNA Extraction, 16S rRNA Gene Amplification, and MiSeq PE300 Sequencing

DNA was extracted from the samples of the four compost manures stored at −20 °C using the FastDNA^®^ Spin Kit for Soil (MP Biomedicals, Santa Ana, CA, USA) according to the manufacturer’s instructions. The DNA in the samples was detected by 1% agarose gel electrophoresis. The V3–V4 hypervariable regions of the bacterial 16S rRNA genes were amplified by PCR using the primers 338F (5′-ACTCCTACGGGAGGCAGCAG-3′) and 806R (5′-GGACTACHVGGGTWTCTAAT-3′). The PCR products were analyzed on a GeneAmp 9700 PCR system (Applied Biosystems, Foster City, CA, USA). Each sample was amplified in triplicate, and the amplified products were mixed and detected by 2% agarose gel electrophoresis. Subsequently, purified amplicons were pooled in equimolar amounts and sequenced using an Illumina Miseq PE300 platform (Majorbio-Biopharm Technology, Shanghai, China).

### 2.3. Data Analysis

To minimize sequencing errors, low-quality sequences (<Q30) were first trimmed out using Trimmomatic 0.35 [22]. The paired reads (with minimum 100-bp overlap) were merged and filtered using PANDAseq [23]. Qualified reads were processed using the Quantitative Insights Into Microbial Ecology pipeline [23]. TrimGalore (http://www.bioinformatics.babraham.ac.uk/projects/trim_galore/ (accessed on 15 July 2020) and Flash [24] were used to process the final V3–V4 tag sequences. Operational taxonomic units (OTUs) were clustered based on a 97% similarity cut-off using UPARSE (version 7.1 http://drive5.com/uparse/ (accessed on 15 July 2020)) using a novel “greedy” algorithm that performs chimera-filtering and OTU-clustering simultaneously [24]. The taxonomy of each 16S rRNA gene sequence was analyzed using the RDP Classifier algorithm (http://rdp.cme.msu.edu/ (accessed on 15 July 2020)) against the Silva 16S rRNA database with a confidence threshold of 70%. The sequence numbers in each sample were normalized to the smallest sample size using the “normalized. shared” command in Mothur [25]. High-throughput sequencing data have been deposited in the National Center for Biotechnology Information Sequence Read Archive (BioProject ID PRJNA723459, study accession number SRP14294735–SRP14294746).

### 2.4. Predatory Myxobacteria Community Abundance

Based on the 16S rRNA gene-based OTU results, a total of 682 OTUs annotated as Myxococcales (myxobacteria) at the order level were selected from all the 26,412 bacterial OTUs clustered. Predatory myxobacteria community abundance information was obtained based on the abundance information for the 682 myxobacteria OTUs.

### 2.5. Statistical Analysis

The α-diversity (Observed OTU richness, Chao1, Shannon, ACE, and Simpson) of myxobacteria and bacteria were estimated using Mothur [26]. The hierarchical cluster tree was calculated using the “vegan” package in R 3.5.1 (R Foundation for Statistical Computing, Vienna, Austria). Based on the abundance of the bacterial and myxobacteria OTUs, principal coordinate analysis (PCoA) and multivariate regression trees (MRT) were performed and constructed using the “vegan” and “mvpart” packages, respectively, in R 3.5.1 (R Foundation for Statistical Computing), Linear regression analysis was used to test the correlation between myxobacteria diversity and bacterial diversity, with sample variety as a random effect, myxobacteria diversity as the dependent variable and bacterial diversity as the independent variable. Analysis of the differences in the abundance of myxobacteria OTUs was performed using the “edgeR” package in R 3.5.1 [27]. Network analysis and LEfSe analysis were generated for network graph visualization using free online platforms, Majorbio I-Sanger Cloud Platform (http://www.i-sanger.com (accessed on 25 July 2020)) and MicrobiomeAnalyst (https://www.microbiomeanalyst.ca (accessed on 25 July 2020)).

Structural equation modeling (SEM) was used to explore the direct and indirect effects of physicochemical parameters and bacterial community diversity on myxobacteria community structure using the “lavaan” package in R 3.5.1 [28]. Our SEM analysis included six variables: myxobacteria diversity, bacterial diversity, pH, TN, Mg^2+^, and Ca^2+^. Distributions of myxococcales in the four types of manure compost were assessed using the “vcd” package in R 3.5.1 and Circos (http://circos.ca/ (accessed on 28 July 2020)). Other statistical analyses were conducted using SPSS 13.0 (SPSS Inc., Chicago, IL, USA).

## 3. Results

### 3.1. Bacteria and Myxobacteria Diversity and Abundance in Different Compost Manures

In the present study, a total of 762,476 high-quality bacterial and 20,210 myxobacteria sequences were obtained after quality control filtering and removal of potential chimeras. The number of bacteria and myxobacteria 16S rRNA sequences per sample ranged from 21,767 to 778,182, and 762 to 2140, respectively. Based on 97% sequence similarity, bacterial and myxobacteria sequences were clustered into 26,412 and 682 OTUs, respectively. Bacterial sequences were primarily composed of the phyla Proteobacteria (32%), Bacteroidetes (25%), Actinobacteria (13%), Chloroflexi (9%), Acidobacteria (8%), and Firmicutes (5.8%) (Figure S1). Conversely, the majority of myxobacteria sequences, in addition to the unassigned OTUs, belonged to the family Blrii41 (80%), Nannocystaceae (8%), Haliangiaceae (6%), Sandaracionaceae (3%), and Polyangiaceae (2%).

**Figure 1:**
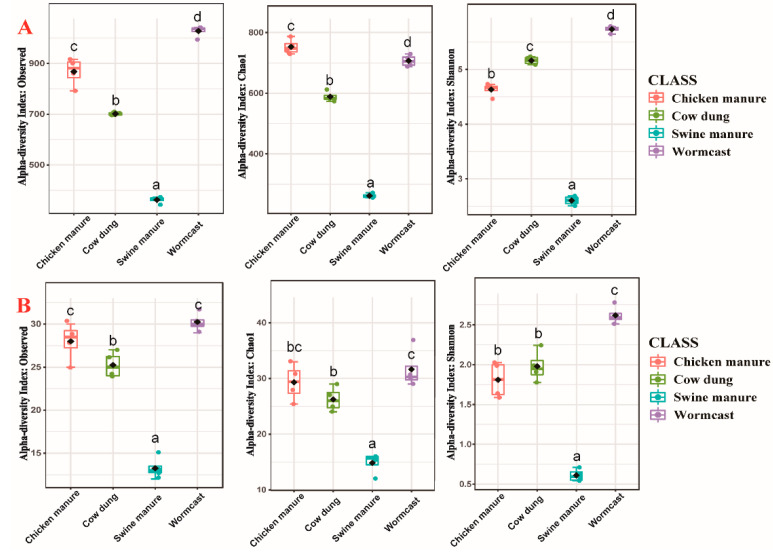
α-diversity of bacteria (**A**) and myxobacteria (**B**) communities in four compost manures. The lowercase letters (a–d) above the box represent significant differences (*p* < 0.05) between the α-diversity at different compost manures.

Alpha-diversity indices (Observed OTU richness, Chao1, and Shannon diversity) values for bacterial and myxobacteria communities in different compost manures are illustrated in Figure 1. One-way Analysis of Variance results showed that the bacterial and myxobacteria community diversity and abundance were significantly different (*p* < 0.05) (Figure 1, Table S1) among four compost manure treatments. The relative abundance and diversity of bacteria differed significantly among the four types of compost manure (*p* < 0.05) (Table S1). In the case of myxobacteria communities, the alpha-diversity indices were the lowest in SM (Observed OTU richness = 13.25, Chao1 = 14.83, Shannon = 0.61), and the highest in WC (Observed OTU richness = 30.25, Chao1 = 31.65, Shannon = 2.62). Although myxobacteria abundance in CM was higher than that in CD, myxobacteria diversity exhibited opposite trends in the two compost manures (Figure 1B). In addition, myxobacteria abundance and diversity trends in all four types of compost manure were similar to those of bacteria (Figure 1A).

### 3.2. Myxobacteria Community Structure among the Four Compost Manures

Based on the relative abundances of bacteria in different compost manures, the main bacterial orders in all samples were Micrococcales, Xanthomonadales, Clostridiales, Anaerolineales, Flavobacteriales, Rhizobiales, Sphingobacteriales, Pseudomonadales, and Myxococcales (Figure S2). Myxococcales was a major taxon, accounting for approximately 3.15% of the total bacteria in the four types of compost manures.

**Figure 2:**
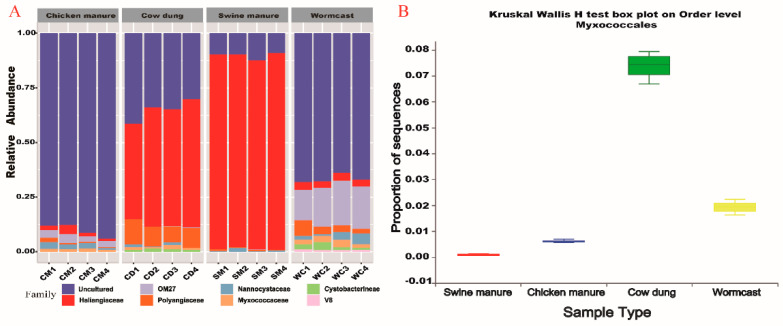
Myxobacteria community composition (**A**) and distribution (**B**) among the four compost manures. CM: Chicken manure, CD: Cow dung, SM: Swine manure, and WC: Wormcast.

At the family level, within the myxobacteria communities, the dominant families (merging small taxa with counts < 10, Figure 2A) across the four types of compost manure were Blrii41 (80%), Nannocystaceae (8%), Haliangiaceae (6%), Sandaracionaceae (3%) and Polyangiaceae (2%). Haliangiaceae abundance in the WC and SM was higher than those in the other two compost manure types. Conversely, BIrii41 relative abundance was the highest in CD, and Nannocystaceae relative abundance was the highest in SM. Biomarkers were bacteria showing significant differences between groups in the LEfSe analysis [29]. BIrii41 was the most active family in CD and Sandaracinaceae was the dominant taxa in CM. Nannocystaceae and Haliangiaceae were the biomarkers in SM and WC, respectively (Figure S3). At the order level, the relative abundance of Myxococcales was significantly different among the four types of compost manure (*p* < 0.05) (Figure 2B, Table S2), while CD had the highest abundance (CD > WC > CM >SM, 74% > 19% > 6.1% > 0.78%).

**Figure 3:**
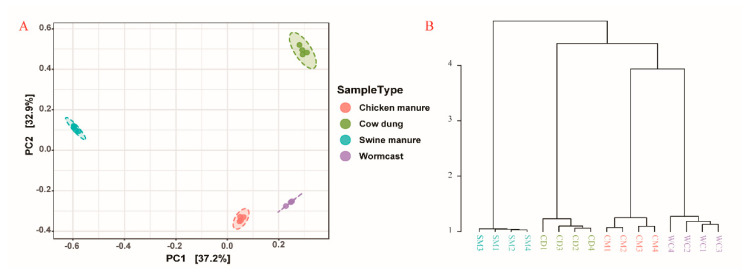
Principal coordinate analysis ((**A**) PCoA) and hierarchical cluster tree (**B**) of myxobacteria communities (calculated based on Bray_Curtis distances). CM: Chicken manure, CD: Cow dung, SM: Swine manure, and WC: Wormcast.

According to the PCoA (carried out based on Bray-Curtis distances) plots (Figure 3A) and Dendrogram Analysis (carried out based on Bray-Curtis distances) results (Figure 3B), myxobacteria community structure was significantly different among the four types of compost manure (*p* < 0.05) (Table S2). In the first component of the PCoA analysis (PCoA1), the community structures in CM and WC were rather similar, and the hierarchical clustering trees showed similar results (Figure 3).

### 3.3. Correlations between Myxobacteria and Bacterial Community Diversity

**Figure 4:**
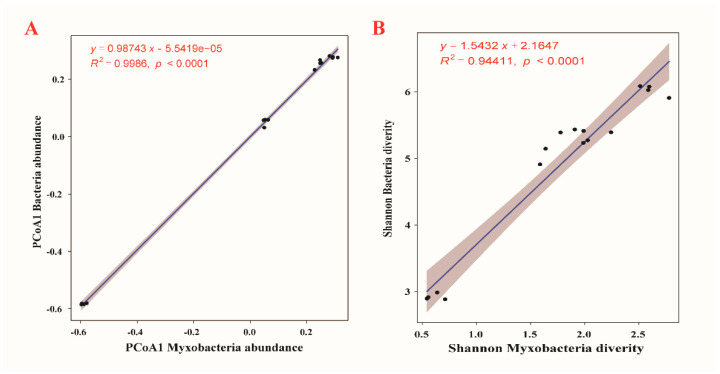
Relationship between myxobacteria and bacterial community abundance (**A**) and diversity (**B**) within four types of compost manure.

The community distribution of myxobacteria in compost manure was correlated with significantly bacterial community diversity. There were significant and positive linear correlations between myxobacteria and bacterial community diversity (α- and β-diversity) in the four compost manures (Figure 4, *p* < 0.0001). The PCoA1 axes of myxobacteria abundance and bacteria abundance showed significant linear relationships with each other (Figure 4A, R^2^ = 0.9986, *p* < 0.0001), and, among the multiple diversity indices, there were consistent results regarding Shannon diversity between myxobacteria and bacteria (Figure 4B, R^2^ = 0.94411, *p* < 0.0001).

### 3.4. Correlation between Myxobacteria Community Diversity and Composition, and Environmental Parameters

**Figure 5:**
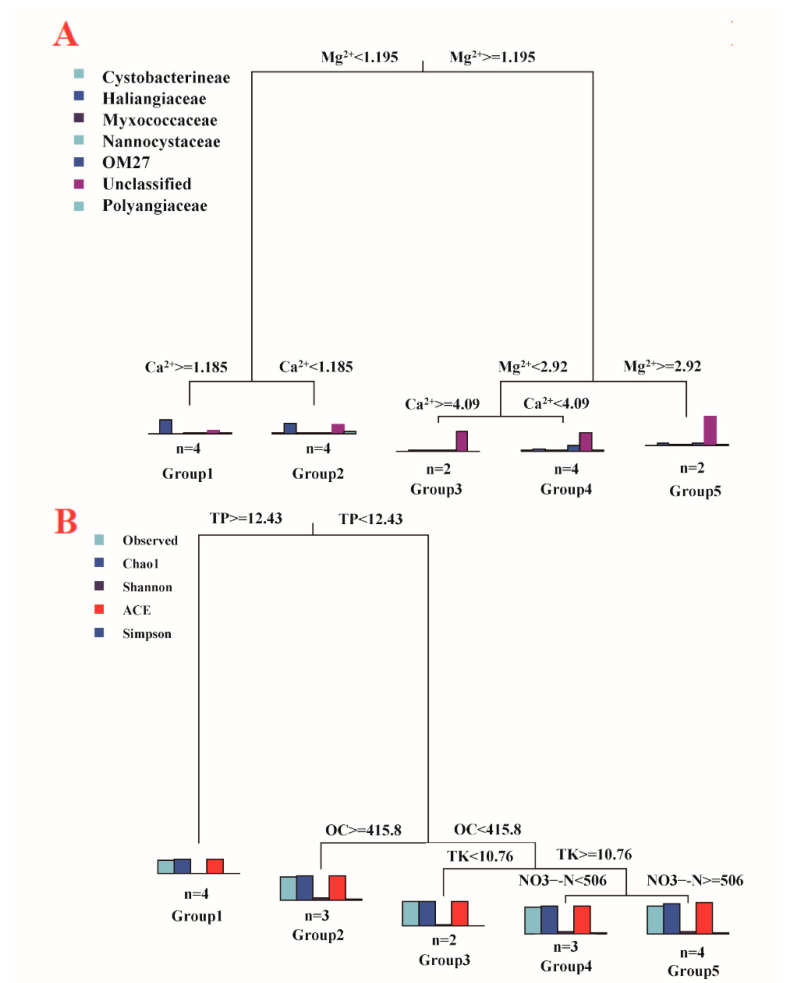
Multivariate regression tree (MRT) analysis of the correlation between myxobacteria composition (**A**) and diversity (**B**), and environmental factors. (**A**): The bar plots illustrate the relative abundance of each order, and the bar plots represent the community composition dynamics among the splits. (**B**): Diversity indices, including Observed OTU richness, Chao1, Shannon, ACE, and Simpson index, standardized for MRT analysis. Bar plots show the multivariate means of diversity among each split. The numbers under the bar are the numbers of samples in each group. TP: total phosphorus, OC: organic carbon, TK: total potassium, NO_3_^−^-N: nitrate nitrogen.

The physicochemical properties of the different compost manure samples are listed in Table 1. To investigate whether there was a relationship between OTU-level myxobacteria community structure and the physicochemical properties of the compost manures, MRT analysis was performed and visualized based on a tree with two splits based on Ca^2+^ and Na^+^ concentrations (Figure 5A). The tree explained 99.16% of the variance in myxobacteria composition among the four types of compost manures (Table S3). The histograms at the three nodes of the tree illustrate an overview of the myxobacteria community structure. Myxobacteria community composition was first split by Ca^2+^ (threshold value 1.16 g·kg^−1^), which explained 98.06% of the variation. The Cluster represents a group of compost manure samples under the split. Cluster 1 and Cluster 2, with twelve manure compost samples, had Ca^2+^ concentrations ≥ 1.16 g·kg^−1^, and the other four compost manure samples in Cluster 3 had Ca^2+^ concentrations < 1.16 g·kg^−1^. Compost manure Na+ concentrations (threshold value 3.2 g·kg^−1^) further split the twelve manure composts samples into two branches and explained 1.1% of the variation. In Cluster 3, the predominant bacterial taxa were BIrii41 (97.85%) and Nannocystaceae (0.15%). Among all the bacterial taxa, the abundance of BIrii41, Nannocystaceae was the most influenced by Mg^2+^ and Na^+^ concentrations in compost manure (Table S4).

The relationship between myxobacteria community diversity and environmental factors were also illustrated based on MRT analysis results, with four splits based on TP, OC, TK, and NO_3_^−^-N (Figure 5B, Table S3). The tree accounted for 95.99% of the variation in the standardized diversity indices. TP split the 16 compost manure samples into two branches with different diversity patterns, including fours samples in Cluster 1 with TP ≥12.43 g·kg^−1^ and 12 samples in Cluster 2, 3, 4, and 5 with TP < 12.43 g·kg^−1^. Samples with relatively low TP (<12.43 g·kg^−1^) had relatively high diversity indices (Observed OTU richness, Chao1, Shannon, ACE, and Simpson). The two branches were further split by OC, and relatively high diversity indices were observed in samples with relatively low OC. Like OC, TP explained 90.61% of the variation. TK and NO_3_^−^-N further influenced bacterial diversity among four compost manures and explained 5.38% of the variation. Overall, the compost manures with relatively high TK and NO_3_^−^-N contents had relatively high diversity indices.

### 3.5. Network Analysis and Structural Equation Modeling of Myxobacteria Community Structure in Compost Manures

**Figure 6:**
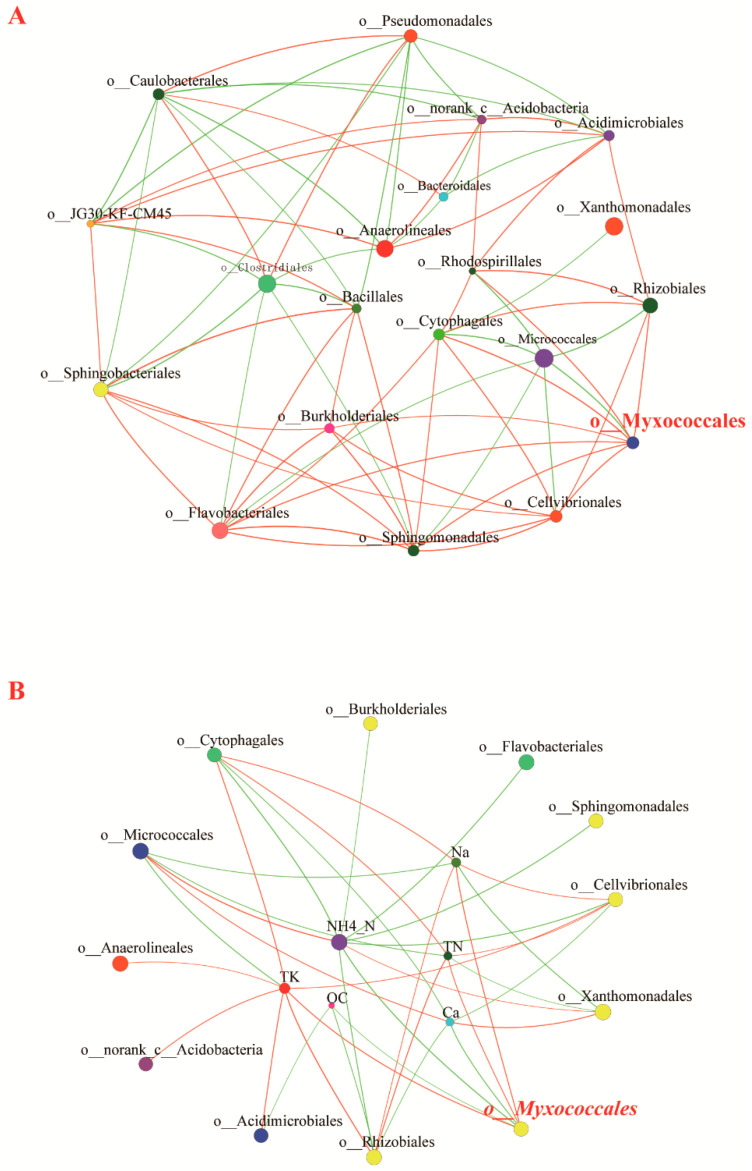
Distribution of predatory myxobacteria in different compost manures. Single (**A**) and two-factor (**B**) correlation network analysis at the order level. A network graph was constructed to illustrate a positive or negative correlation between different bacterial orders. A red link indicates a positive correlation between two individual nodes, whereas a green link indicates a negative correlation. Different nodes represent different bacteria orders or environmental factors. TN: total nitrogen, OC: organic carbon, TK: total potassium, NH_4_^+^-N: ammonium nitrogen.

An ecological network illustrates the correlation of various organisms in an ecosystem. In the correlation network in the present study, the co-occurrence relationship between myxobacteria and other bacteria drove the ecological network topology. As illustrated in Figure 6A, a single factor correlation network with 20 nodes was constructed based on the four types of compost manure under study (Table S5). In the network, myxobacteria and other bacteria (order level) formed a complex topological network structure (absolute value of Spearman’s correlation coefficient ≥ 0.6). The Myxococcales node had a relatively high degree and clustering coefficient, and co-occurred with some nutrition-related bacteria; Myxococcales had a significant and positive correlation with bacterial orders (Cellvibrionales, Sphingomonadales, Flavobacteriales, Burkholderiales, Cytophagales, Rhodospirillales, and Rhizobiales), and a significant and negative correlation with Micrococcales (Figure S4).

According to the results of the two-factor correlation network analysis, the abundance of myxobacteria was significantly related to various environmental factors. In the four types of compost manure, Na^+^, TN, and TK concentrations were significantly and positively correlated with Myxococcale abundance; conversely, OC, Ca^2+^, and NH_4_^+^-N concentrations were significantly and negatively correlated with Myxococcale abundance, which is consistent with the results of the MRT analysis.

**Figure 7:**
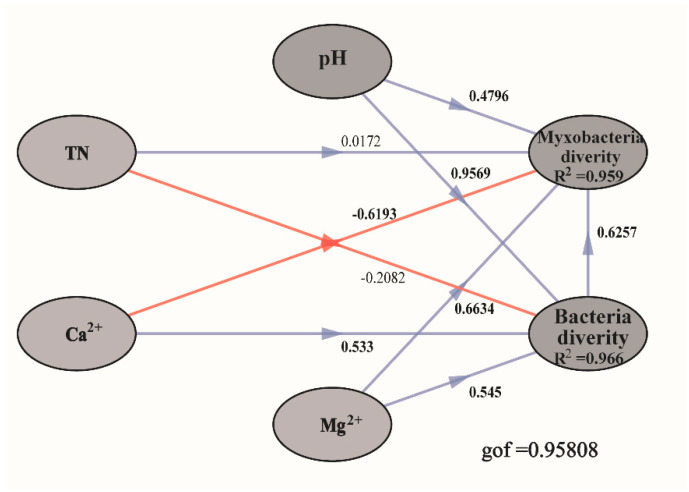
Structural equation models (SEMs) showing the effects of pH, total nitrogen (TN), Calcium ions (Ca^2+^), Magnesium ions (Mg^2+^), and bacterial diversity on myxobacteria diversity. Values on the arrows represent the path coefficients. Red and blue lines indicate the significant (*p* < 0.05) and non-significant (*p* > 0.05) relationships, respectively. The “gof” indicates the goodness of fit. R^2^ denotes the proportion of variance explained. Bacterial and myxobacteria community diversity is represented using α-diversity indices (Observed OTU richness, Chao1, and Shannon).

According to the SEM results (Figure 7), bacterial diversity, Mg^2+^ concentrations, and pH positively influenced myxobacteria diversity, while Ca^2+^ concentration had an opposite effect. In addition, metal ions had potentially varied effects on the diversity of different microorganisms in compost manure. For example, Ca^2+^ had a positive effect on bacterial diversity and a negative effect on myxobacteria diversity. Overall, our model explained 95.9% of the variation in myxobacteria diversity.

## 4. Discussion

Soil bacteria biogeography could reveal significant correlations between bacterial community distribution and environmental factors [30]. Moreover, microorganism distribution and development are influenced by complex interactions with plants, animals, and other microbes, which could have beneficial, neutral, or harmful effects on bacterial community members [6]. In the present study, we investigated the community profile of predatory myxobacteria in different compost manures. Overall, the results indicated that bacterial community diversity, Mg^2+,^ and Ca^2+^ concentrations, and pH were associated with myxobacteria community diversity in different compost manures.

### 4.1. Myxobacteria Community Structure in Different Compost Manures

Myxobacteria are mainly distributed in soil environments and most predatory myxobacteria are isolated from agricultural soils [2]. Compared with conventional farming, organic farming with organic fertilizer amendment can enhance microbial diversity and richness [19]. The application of organic fertilizer can significantly increase the diversity and richness of myxobacteria in the soil [20]. According to a previous study, myxobacteria in a single soil sample accounted for 4.1% of the entire bacterial community [21], and Myxococcales sequences accounted for 1.31−4.17% of the sequences in 16S rRNA gene libraries in the four types of compost manure examined in the present study. The relative abundance of myxobacteria in farmland soils [19] and subtropical and tropical forest soils [6] have been reported to account for 0.36–4.10% and 1.49–4.74% of the total bacterial abundance, respectively, which are consistent with the results reported in the present study under compost manure.

Differences in the type of compost manure markedly affected the structure of myxobacteria communities at the family level. Myxobacteria were unevenly distributed in different compost manures, and not all myxobacteria families could be observed in the samples examined. In the LEfSe analysis (Figure S3), BIrii41, Nannocystaceae, Sandaracinaceae, and Haliangiaceae were prominent microorganisms in different compost manures. Previous studies showed that BIrii41 was abundant in aerobic compost and vermicompost [31]. Most members of Nannocystaceae are regarded as halotolerant and halophilic organisms capable of degrading complex macromolecules and lysing microorganisms, and Haliangiaceae represent bacteriolytic- and non-cellulolytic-type obligate halophilic myxobacteria, and currently the only known application is the production of novel biologically active compounds [32]. The species of Sandaracinaceae are heterotrophic consumers of low-molecular-weight organic compounds, such as ethanol, hydrogen, butyrate, and acetate [33]. In our study, salt ions may play indispensable roles in the specific myxobacteria taxa substantially enriched among four compost manures. In addition, myxobacteria diversity in WC was significantly higher than that in SM. This could be because compared to SM, WC contains more easily usable organic substances [34], and higher microbial diversity and abundance [35], which provides adequate food and a suitable environment for myxobacteria development and survival.

### 4.2. Effects of Abiotic Factors on Myxobacteria Community Structure in Compost Manure

According to previous reports, microbial community structure is mainly influenced by environmental factors [36], and soil characteristics are correlated with myxobacteria abundance [21]. Similarly, myxobacteria distribution in the different compost manures in the present study was influenced by abiotic factors. We also observed correlations between abiotic factors of compost manure and myxobacteria community structure. In addition, according to the SEM results, abiotic factors of manure compost influenced myxobacteria community structure directly.

According to the results of the present study, abiotic factors significantly influence the community profile of predatory myxobacteria in different compost manures. Specifically, pH, TN concentration, and Mg^2+^ concentration are significantly positively correlated with myxobacteria community diversity; conversely, Ca^2+^ and NH_4_^+^-N concentrations in compost manure were significantly and negatively correlated with myxobacteria community diversity. pH is the major abiotic factor influencing the distribution of microorganisms in different environments [37,38,39,40]. Similarly, pH considerably influenced myxobacteria distribution in compost manure in the present study. Myxobacteria are mostly distributed in environments with a pH of approximately 6.5–8.5, especially in neutral to weakly alkaline soils with pH 6.0–8.0 [41,42]. Excluding in the case of CD (pH 9.21–9.47), the pH values of the other three compost manures (pH 7.09–7.82) were all within the optimal range for the myxobacteria survival, which could explain the high myxobacteria abundance in the three compost manures. NH_4_^+^-N is the preferred nitrogen source for most microorganisms [43]. Therefore, NH_4_^+^-N could positively influence bacterial community structure when agricultural waste compost is adopted as fertilizer [36]; however, in the present study, we observed that myxobacteria abundance in compost manure was significantly negatively correlated with NH_4_^+^-N concentration, which is similar to results on the distribution of predatory bacteria in soil [20].

Salt ion concentrations can influence the growth and development of myxobacteria [44,45,46,47]. Mg^2+^ and Ca^2+^ are generally considered to promote myxobacteria development [48,49]. In the present study, high Ca^2+^ concentrations were the major reason for the decrease in myxobacteria diversity in the four types of compost manure, with Ca^2+^ concentration being negatively correlated with myxobacteria diversity; however, it can positively affect bacterial diversity in the four types of compost manure. In addition, the complex interactions between myxobacteria and indigenous microorganisms cannot be overlooked. High concentrations of Ca^2+^ could promote the growth of some bacteria antagonistic to myxobacteria and, in turn, influence myxobacteria diversity. According to our results, high Ca^2+^ concentrations were significantly and positively correlated with Micrococcales abundance, while Micrococcales abundance was significantly and negatively correlated with Myxococcales abundance and diversity, which is partly consistent with our speculation.

### 4.3. Effect of Manure Compost Bacterial Diversity on Myxobacteria Community Structure

Microbial interactions such as auxotrophies and nutrient demands among members of a microbial community are key drivers of microbial community structure [50]. As a major class of predatory bacteria in microbial communities, myxobacteria can prey on Gram-negative and Gram-positive bacteria, yeasts, fungi, protozoa, and nematodes [2,51]. We observed a significant correlation between myxobacteria diversity and bacterial community composition under different compost manures. Although no causal relationship was established, the results revealed a direct correlation between potential prey and predators at the community level.

Because soil myxobacteria cannot synthesize riboflavin and branched-chain amino acids [3], their community structures could be influenced by prey availability. In the present study, there was a positive correlation between the relative abundance of bacteria and the relative abundance of myxobacteria in compost manure. Considering predation of myxobacteria on prey bacteria, the reason for the increase in myxobacteria abundance is potentially an increase in the number of prey bacterial cells. Notably, some researchers have reported that *Corallococcus* sp. EGB strains control cucumber wilt disease by migrating to plant roots and regulating soil microbial community structure at the sites [12]. Consistent with our results, other studies on predator–prey diversity relations have reported a positive correlation between predator abundance and prey abundance [52,53,54]. To the best of our knowledge, this is the first study to explore the relationship between predatory myxobacteria and bacterial community structure across different types of compost manure.

Preferential predation by micro-predators could explain the influence of bacterial communities on predatory microbial community structure. According to the results of the single-factor correlation network analysis in the present study, in the microbial correlation network of the different compost samples, Myxococcales were significantly positively correlated with Sphingomonadales, Flavobacteriales, Cellvibrionales, Cytophagales, Burkholderiales, Rhizobiales, and Rhodospirillales (order level), which implies potential predator–prey relationships. The Gram staining characteristics of prey bacteria could influence prey selection by myxobacteria. Myxococcus are reportedly more supported by Gram-negative prey than by Gram-positive bacteria [55]. We also note that in the present study, Myxococcales nodes in the correlation network were significantly and positively correlated with Gram-negative bacteria, and significantly and negatively correlated with Gram-positive bacteria (Micrococcales). However, the overall conclusion that Gram-negative prey can more effectively support Myxococcales requires further investigation and evidence. Other studies have also reported that Haliangiaceae are effectively supported by Arthrobacter globiformis (a Gram-positive actinomycete) [5]. In the present study, we highlight the influence of bacterial community structure on myxobacteria community profile in different compost manures. Nevertheless, the key bacterial taxa driving the distribution patterns of myxobacteria require further investigations.

## 5. Conclusions

In the present study, we reveal the key factors influencing myxobacteria distribution in different compost manures for the first time. We report that abiotic factors (pH and Mg^2+^) have positive effects on myxobacteria community diversity as well as bacterial community diversity. However, high Ca^2+^ concentrations have negative effects on myxobacteria diversity. Overall, bacterial community diversity and Mg^2+^ and Ca^2+^ were the major factors influencing myxobacteria distribution in different compost manures. Nevertheless, due to the complex predator–prey interactions, our data failed to determine the specific bacterial groups influencing the myxobacteria distribution in the compost manures. Our findings could facilitate the selection of appropriate compost manure types and appropriate management of soil management strategies based on the physicochemical properties of the compost, which could not only increase myxobacteria community diversity and abundance in manure compost but also enhance soil health in farmland amended with organic fertilizer.

## Figures and Tables

**Table 1:** Physicochemical characteristics of the four types of compost manures. OC, organic carbon; TN, total nitrogen; TP, total phosphorus; TK, total potassium; NO_3_^−^-N, nitrate nitrogen; NH_4_^−^-N, ammonium nitrogen; Ca^2+^, Calcium ions; Mg^2+^, Magnesium ions; Na^+^, Sodium ions. CM, chicken manure; SM, swine manure; CD, Cow dung; WC, wormcast.

Physicochemical Property	Chicken Manure	Swine Manure	Cow Manure	Wormcast
pH	7.165 ± 0.061	7.505 ± 0.153	9.305 ± 0.116	7.77 ± 0.081
OC g·kg^−1^	196.485 ± 0.427	281.715 ± 0.244	416.285 ± 0.728	127.27 ± 0.129
TN g·kg^−1^	21.348 ± 0.134	24.108 ± 0.90	21.693 ± 0.102	13.293 ± 0.22
TP g·kg^−1^	9.7475 ± 0.195	15.013 ± 0.013	4.3425 ± 0.033	9.305 ± 0.013
TK g·kg^−1^	10.75 ± 0.084	11,233 ± 0.78	12.295 ± 0.465	17.51 ± 0.259
NO_3_^−^-N g·kg^−1^	1078.05 ± 5.98	6.105 ± 0.183	4.78 ± 0.143	506.115 ± 1.96
NH_4_^+^-N g·kg^−1^	282.23 ± 4.224	8175.23 ± 8.517	92.818 ± 0.637	143.305 ± 2.04
Ca^2+^ g·kg^−1^	6.21 ± 0.075	2.07 ± 0.097	0.4125 ± 0.033	1.9825 ± 0.108
Mg^2+^ g·kg^−1^	2.92 ± 0.034	1.5175 ± 0.041	0.995 ± 0.037	2.3525 ± 0.073
Na^+^ g·kg^−1^	2.0275 ± 0.122	2.61 ± 0.265	12.688 ± 0.143	3.83 ± 0.081

## Data Availability

All data generated or analyzed during this study are included in this published article and its supplementary information files.

## Supplementary Materials

The following are available online at https://www.mdpi.com/article/10.3390/microorganisms9112193/s1 Figure S1: The relative abundance of bacteria under different compost manure treatments; Figure S2: Circos plot showing Myxobacteria community abundance and composition under different compost manure treatments; Figure S3: The LEfSe method identified significant differences among communities with different compost manures; Figure S4: Heatmap plot showing correlation of dominant bacterial order under different compost manure treatments; Table S1: One-way ANOVA analysis of physicochemical properties and microbial community diversity under different compost manure treatments; Table S2: PERMANOVA analysis of ccommunity under different compost manure treatments; Table S3: Contributions of compost parameters to the myxobacteria community composition and diversity (multivariate regression trees analysis); Table S4: Myxobacteria relative abundance for each split; Table S5: Single factor correlation network node attribute table.
