# Supplementary material for: Community Profile and Drivers of Predatory Myxobacteria under Different Compost Manures
Source: Microorganisms. 2021 Oct 21;9(11):2193. doi: 10.3390/microorganisms9112193 (PMC8622275; doi:10.3390/microorganisms9112193)
Supplement: Supplementary file 1 [file microorganisms-09-02193-s001.zip › microorganisms-1406718-supplementary.pdf]

Supplementary

Supplementary Figures and Tables

Table S1.

Table S1 One-way ANOVA analysis of physicochemical properties and microbial community diversity under different compost manure treatments.

Variables	Phase	
	F	P
OC	313949.5	<0.001***
NO_3^--N	105678.5	<0.001***
NH_4^+-N	2698533	<0.001***
TN	11329.87	<0.001***
C/N	25.93	<0.001***
TK	26621.98	<0.001***
pH	8.585	0.003**
TP	11460.81	<0.001***
Ca^{2+}	6373.427	<0.001***
Mg^{2+}	1703.392	<0.001***
Na^+	6714.4	<0.001***
Bacterial Shannon index	823.659	<0.001***
myxobacteria Shannon index	101.905	<0.001***
Bacterial Chao1 index	170.891	<0.001***
myxobacteria Chao1 index	27.214	<0.001***
myxobacteria abundance	3.179	0.063
Bacterial abundance	6.993	0.006**

Note: OC, organic carbon; NO_3^--N, nitrate nitrogen; NH_4^+-N, ammonia nitrogen; TN, total nitrogen; TP, total phosphorus; C/N, total organic carbon / total nitrogen; TK, total potassium; Ca^{2+}, Calcium ion; Mg^{2+}, Magnesium ions; Na^+, Sodium ion. ***$P < 0.001$; **$P < 0.01$; *$P < 0.05$

Table S2.

Table S2 PERMANOVA analysis of ccommunity under different compost manure treatments.

Group	Df	Sums of squares	Mean squares	F.Model	Variation (R2)	Pr (>F)	Sig
CM/SM	1	1.7593	1.7593	154.9796	0.963	0.025	*
CM/CD	1	1.7010	1.7010	65.8035	0.916	0.025	*
CM/WC	1	1.4227	1.4227	45.8357	0.884	0.031	*
SM/CD	1	1.8912	1.8912	103.8493	0.945	0.032	*
SM/WC	1	1.8591	1.8591	79.4450	0.930	0.031	*
CD/WC	1	1.6570	1.6570	43.7226	0.879	0.023	*

Note: CM, chicken manure; SM, swine manure; CD, Cow dung; WC, wormcast. ***P < 0.001; **P < 0.01; *P < 0.05

Table S3.

Table S3 Contributions of compost parameters to the myxobacteria community composition and diversity (multivariate regression trees analysis).

Branch condition	Branch point (%)			
	Ca^{2+} 1.16 g·kg-1	Na^{2+} 3.2g·kg-1	col_total_tree	col_total_species
BIrii41	97.84916	0.338304407	98.187462	98.90377
Nannocystaceae	0.1471139	0.114221722	0.2613356	0.2797915
Haliangiaceae	0.049495	0.562034082	0.6115290	0.6425704
sandaracinaceae	5.27335E-05	0.024308855	0.0243615	0.07960629
polyangiaceae	0.01129674	0.048546237	0.0598429	0.06605072
Archangiaceae	0.002651495	0.008032931	0.0106844	0.02145776
phaselicystidaceae	0.002517264	0.001844366	0.0043616	0.006756866
total_col	98.06229	1.097292599	99.159578	100

Branch condition	Branch point (%)					
	TP 12.43 g·kg-1	OC 415.8 g·kg-1	TK 10.76 g·kg-1	NO_3-N 506 mg·kg-1	col_total_tree	col_total_species
Observed	28.0193	1.7615	0.6444	0.1887	30.614	31.5509
Chao1	26.7229	2.4597	1.4703	0.941	31.5939	33.6073
Shannon	0.3065	0.0105	0.0185	0.0095	0.345	0.3849
ACE	28.8361	2.4563	1.224	0.8819	33.3983	34.4143
Simpson	0.0363	0.0008	0.0016	0.0008	0.0394	0.0425
Total_col	83.9211	6.6888	3.3589	2.0219	95.9908	100

Table S4

Table S4 Myxobacteria relative abundance for each split.

Split condition	Split 1		Split 2	
	Ca<1.16 g/kg	Ca≥1.16 g/kg	Na≥3.2 g/kg	Na<3.2 g/kg
BIrii41	0.010	0.990	0.062	0.928
Nannocystaceae	0.098	0.902	0.372	0.530
Haliangiaceae	0.095	0.905	0.763	0.142
sandaracinaceae	0.353	0.647	0.385	0.262
polyangiaceae	0.113	0.887	0.481	0.406
Archangiaceae	0.359	0.641	0.521	0.120
phaselicystidaceae	0.194	0.806	0.333	0.472
n	4	12	4	8

Table S5

Table S5 Single factor correlation network node attribute table.

node name	degree	clustering
o__Sphingomonadales	9	0.611111
o__Sphingobacteriales	9	0.583333
o__Flavobacteriales	9	0.611111
o__Caulobacterales	9	0.638889
**o__Myxococcales**	8	0.678571
o__Acidimicrobiales	8	0.535714
o__Bacillales	8	0.642857
o__Pseudomonadales	8	0.714286
o__Clostridiales	8	0.642857
o__Cellvibrionales	8	0.678571
o__JG30-KF-CM45	8	0.714286
o__Cytophagales	8	0.571429
o__Anaerolineales	7	0.761905
o__Micrococcales	7	0.761905
o__norank_c__Acidobacteria	7	0.666667
o__Burkholderiales	6	0.8
o__Rhizobiales	6	0.666667
o__Rhodospirillales	6	0.533333
o__Bacteroidales	4	1
o__Xanthomonadales	1	0

Note: Node name is the name of the node in the network graph; Degree represents the degree of the node in the network graph, that is, the number of nodes connected to a node; Clustering is the clustering coefficient of the node, which represents the connection between a node and its neighboring nodes.

Figure S1.

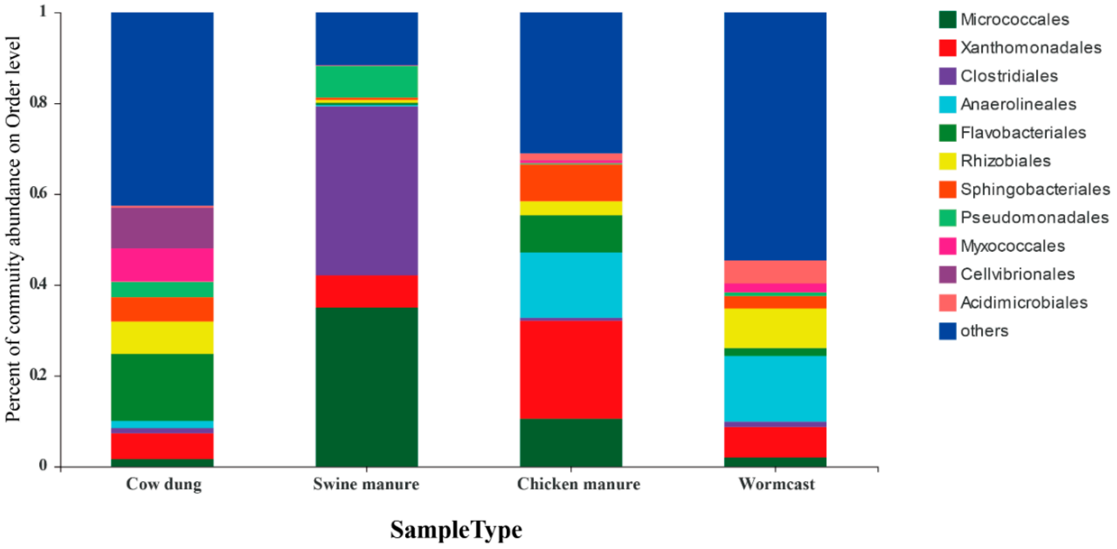

Figure S1. The relative abundance of bacteria under different compost manure treatments.

Figure S2.

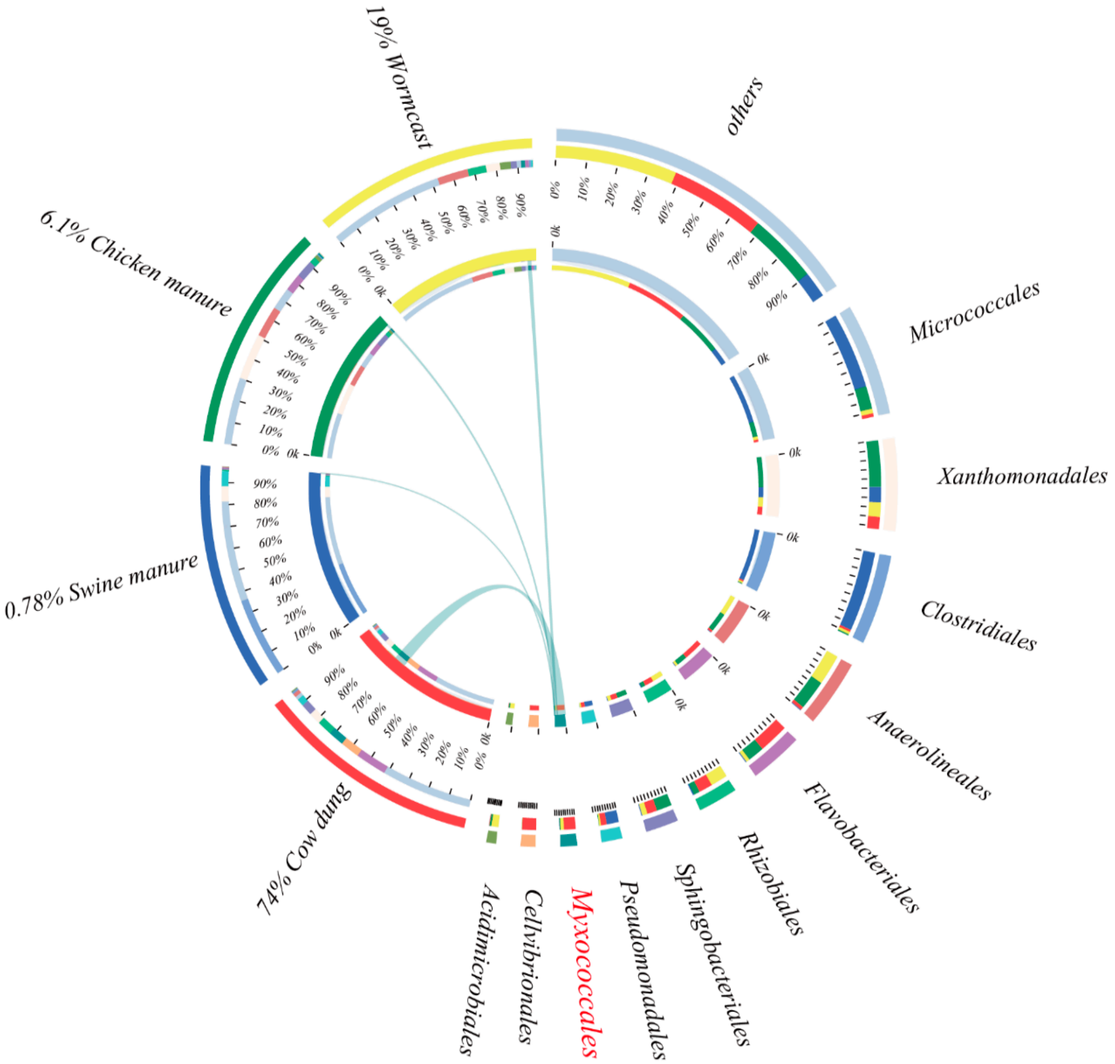

Figure S2. Circos plot showing Myxobacteria community abundance and composition under different compost manure treatments.

Figure S3.

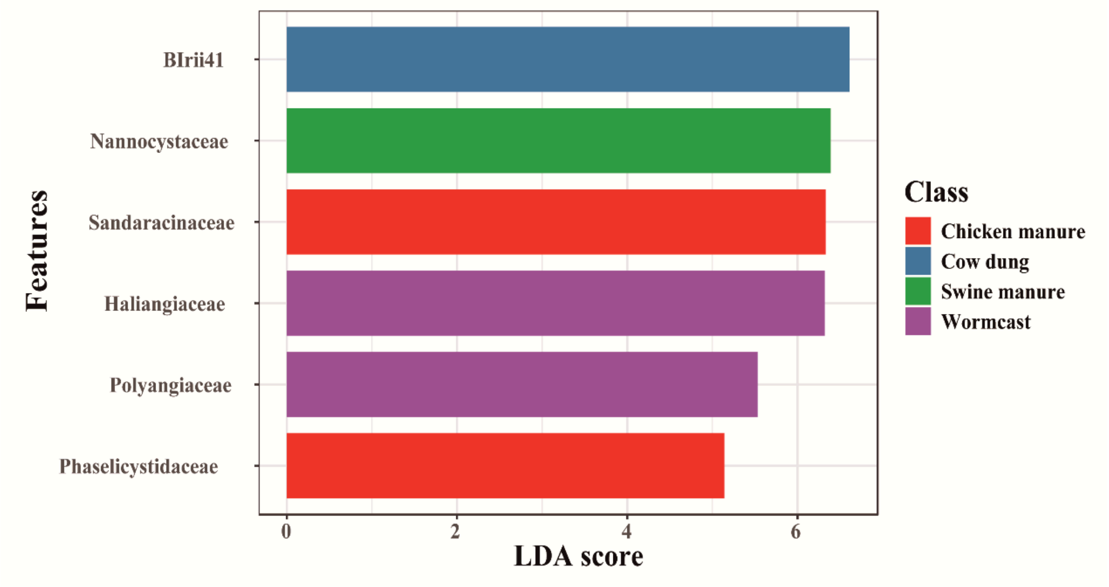

Figure S3. The LEfSe method identified significant differences among communities with different compost manures. Taxonomic units with significant differences ($P < 0.05$) in abundance between compost manures are represented by color.

Figure S4.

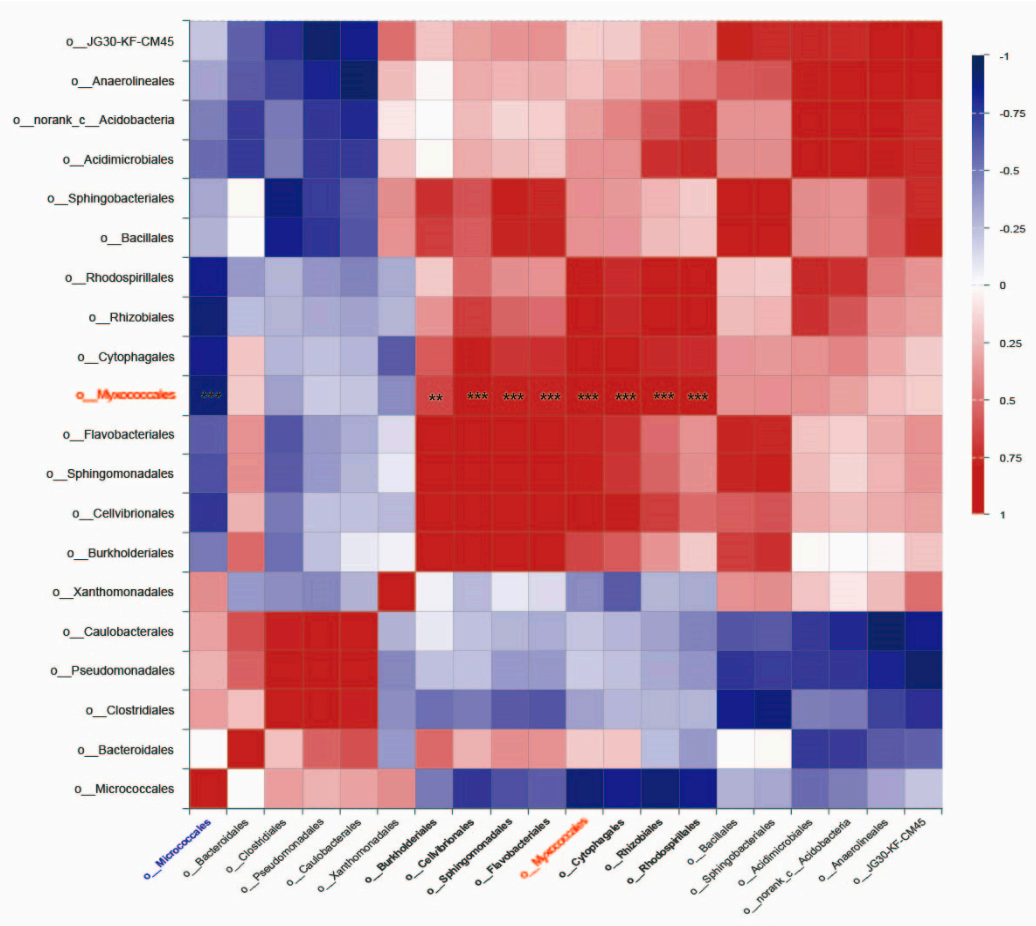

Figure S4. Heatmap plot showing correlation of dominant bacterial order under different compost manure treatments. Note: ***P < 0.001; **P < 0.01; *P < 0.05
